# Temporal asynchrony and spatial perception

**DOI:** 10.1038/srep30413

**Published:** 2016-07-27

**Authors:** Maria Lev, Uri Polat

**Affiliations:** 1 Tel-Aviv University, Faculty of Medicine, Goldschleger Eye Research Institute, Sheba Medical Center, Tel-Hashomer, Israel; 2 School of Optometry and Vision Science, Faculty of life Science, Bar-Ilan University, Ramat-Gan, Israel

## Abstract

Collinear facilitation is an enhancement in the visibility of a target by laterally placed iso-oriented flankers in a collinear (COL) configuration. Iso-oriented flankers placed in a non-collinear configuration (side-by-side, SBS) produce less facilitation. Surprisingly, presentation of both configurations simultaneously (ISO-CROSS) abolishes the facilitation rather than increases it - a phenomenon that can’t be fully explained by the spatial properties of the target and flankers. Based on our preliminary data and recent studies, we hypothesized that there might be a novel explanation based on the temporal properties of the excitation and inhibition, resulting in asynchrony between the lateral inputs received from COL and SBS, leading to cancelation of the facilitatory component in ISO-CROSS. We explored this effect using a detection task in humans. The results replicated the previous results showing that the preferred facilitation for COL and SBS was abolished for the ISO-CROSS configuration. However, presenting the SBS flankers, but not the COL flankers 20 msec before ISO-CROSS restored the facilitatory effect. We propose a novel explanation that the perceptual advantage of collinear facilitation may be cancelled by the delayed input from the sides; thus, the final perception is determined by the overall spatial-temporal integration of the lateral interactions.

Collinear flankers (COL) enhance the visibility of a local target (Gabor patch) that is placed between them^1^. Such detection facilitation is found when the low-contrast target is presented simultaneously with or after high-contrast collinear flankers (see the example in each figure)^1^,^2^,^3^,^4^,^5^,^6^,^7^,^8^,^9^,^10^,^11^,^12^,^13^. Lateral interactions are both excitatory and inhibitory^1^,^8^,^13^,^14^,^15^,^16^,^17^,^18^,^19^,^20^,^21^,^22^,^23^,^24^,^25^, suggesting that they are mediated by the long-range horizontal connections formed by pyramidal neurons within layers 2–3^17^,^18^,^19^,^22^,^26^,^27^,^28^. However, later results suggest that the long-range layer 4 circuitry plays a different functional role than that of iso-orientation biased layers 2–3^29^. It was also found that feedback from higher cortical areas may also play a role^30^.

Facilitative and suppressive center-surround interactions may be organized differently to subserve different functional roles. Facilitative interactions may be organized mainly along the neuron’s optimal orientation, forming a collinear integration field (mirroring the psychophysical “association field” for collinear contour perception)^1^,^6^,^7^,^22^,^31^,^32^,^33^,^34^. Suppression is a more general phenomenon that is found for many center-surround combinations of spatial configurations, orientations, and spatial separations. It was suggested that suppression may have different functions, depending on the contextual parameters^35^.

Studies using single-unit^14^,^16^,^17^,^21^,^31^,^33^,^36^, intracellular^22^,^37^,^38^,^39^, and imaging techniques^40^,^41^,^42^,^43^,^44^ showed that neuronal response is modulated by signals from outside the RF. Since lateral interactions are both excitatory (E) and inhibitory (I)^1^,^8^,^13^,^14^,^15^,^16^,^17^,^18^,^19^,^20^,^21^,^22^, the final outcome, suppression, or facilitation depends on the E/I balance. Although usually it was found that E/I were well balanced^45^, the balance depends on complex spatio-temporal parameters. In the spatial domain, the spatial configuration of the target and surround while in the collinear configuration results mainly in facilitation, whereas non-collinear configurations result in suppression^22^,^34^. The strength of the effect depends on the distance between the target and the surround^22^,^34^ and the contrast of the target and surround; however, low target contrast (mainly in the collinear configuration) reveals facilitation, whereas high contrast reveals suppression^32^,^33^,^35^,^46^,^47^. In the temporal domain, it was found that the propagation of the lateral input is slow^8^,^13^,^22^,^34^,^38^,^48^,^49^, the delay increases with increasing separation between the target and the surround^13^,^22^,^38^,^49^ and that it depends on the global arrangement of the center and surround, being faster for the collinear configuration^22^,^34^,^50^,^51^,^52^. Moreover, the E/I balance may be modified by training^47^,^53^,^54^.

Thus, the emerging results regarding the spatio-temporal properties of excitation and inhibition suggest that the inhibition results from both local^3^,^22^,^33^,^35^,^47^,^55^ and from lateral interactions^22^,^55^, whereas the lateral inhibition is mainly from iso-oriented receptive fields, having strong, transient, and fast time constants, probably via shunting inhibition^22^,^55^. The excitation also derives from lateral interactions predominantly from iso-oriented receptive fields^3^,^22^,^33^,^35^,^47^,^55^,^56^; however, the excitation is sustained and has a slower time constant^8^,^13^,^22^,^33^,^47^. Thus, the E/I level is determined by the spatio-temporal parameters in the network; hence, the level is not influenced by whether the output is suppressive or facilitative^3^,^22^,^33^,^46^,^47^.

An alternative view of the facilitatory effect suggests that the high-contrast flankers directly stimulate neurons that are involved in detecting the target (due to overlapping receptive fields) and thus act as a low-contrast pedestal^57^.

Several studies have demonstrated that non-collinear iso-oriented flankers positioned at the side (side-by-side, SBS) can also facilitate detection^3^,^7^,^13^,^58^, but mostly less than COL. Surprisingly, presenting COL and SBS configurations simultaneously, producing a cross configuration (ISO-CROSS) abolishes the facilitation rather than increases it^35^. Another study found that the arrangement of Gabor elements surrounding the target cancels the facilitatory effect^10^. Taken together, the cancelation effect of collinear facilitation was rather surprising and is difficult to explain by the lateral excitation or large receptive field underlying the facilitatory effect^10^,^35^.

Another possible explanation, still in the spatial domain, is based on the results of recent Yes/No experiments exploring the collinear facilitation. When observers were asked to report the presence (yes) or absence (no) of a near threshold target (a Gabor patch), both their hit rate (reporting “yes” on the target present trials) and their false alarm rate (reporting “yes” on the target absent trials) increase in the presence of nearby collinear flankers^9^,^47^,^59^,^60^,^61^. It was suggested that the hit rate reflects the collinear facilitation and that the false-alarms mimic the “filling-in effect”^47^,^59^,^60^. One interpretation for the filling-in effect is that collinear flankers produce neuronal activity, via lateral interactions, at locations corresponding to the target even if it is not directly activated by feed forward input^9^,^40^,^47^. A recent study^62^ uses an equivalent noise approach to explore the relationships between noise and false alarms. The results are consistent with the notion that nearby collinear flankers add both signal and noise to the target location. The increased signal results in higher hit rates; the increased noise results in higher false alarm rates (the filling-in effect). Thus, in considering the ISO-CROSS configuration, one would expect to find an increased filling-in effect (a false alarm rate), thus increasing the “noise”; hence, it may cancel the facilitation. In this study we explored possible explanations for the cancelation effect using a Yes/No paradigm.

Crowding, the inability to recognize objects in a clutter, sets a fundamental limit on conscious visual perception and object recognition^63^,^64^. Several studies^65^,^66^ used the ISO-CROSS configuration to explore the crowding effect and the relationships between masking and crowding, demonstrating reduced target identification, thus suggesting that masking and crowding are not related. However, our recent spatiotemporal model^47^, which is based on the properties of lateral interactions, posits that masking and crowding are related in the spatial and temporal domains. The results suggest that under certain conditions, crowding and masking share common neural mechanisms that underlie the spatiotemporal properties of excitation and inhibition. Thus, transforming the facilitation in COL to “no-effect” in ISO-CROSS might be due to a shift in the neuronal output responding to the different spatial combinations that lead to different spatiotemporal outputs of excitation and inhibition.

Temporal information can lead to segregation of objects from their background with a time difference within 5 msec^67^,^68^,^69^,^70^,^71^,^72^. It was suggested that binding visual features into a coherent percept consists of synchronizing the activity of their neural representations. The results indicate that a small temporal asynchrony, below the visual integration timescale, can have a direct effect on grouping^73^. The results indicate that visual grouping is indeed facilitated when elements of one percept are presented at the same time as others and are temporally different from elements of another percept or from background elements. The authors concluded that the results indicate that binding is due to a global mechanism of grouping caused by synchronous neural activation^73^. It was largely assumed that collinear interactions serve as mechanisms involved in grouping contour elements^7^,^33^,^35^,^74^,^75^,^76^,^77^,^78^. Although spatial similarity is a fundamental rule in grouping, Polat^35^ suggested that temporal similarity is an additional important rule for grouping. This suggestion was backed by data showing that under collinear facilitation the neural response variance decreases^79^, which consequently increases the temporal correlation between the contour elements^80^. Recent electrophysiological studies^22^,^49^ support the notion of the importance of temporal matching between the feedforward and lateral signals, suggesting that temporal matching is critical for reaching final behavioral relevance such as grouping.

Indeed, there are some indications that the “ISO-CROSS phenomenon” might also be influenced by temporal factors. Several studies have shown that temporal processing of COL and SBS are different^22^,^34^,^50^,^51^,^52^,^72^. It was shown that the direction of motion produces bias in the neural response and in human perception. Using intra-cellular recordings from receptive fields in V1 of cats, it was found that there is a shorter delay when the lateral activation was in the collinear direction than in the orthogonal direction^22^,^34^,^50^. An optical imaging study in V1 of monkeys found that the cortical activity, as measured by the onset synchronization, elicited by collinear flankers, preceded that elicited by orthogonal flankers^40^. MEG recordings in humans showed that the neural latency associated with apparent motion in the direction of co-aligned Gabor elements (mimicking a COL configuration) was faster than the latency of motion in the orthogonal direction (mimicking a SBS configuration)^52^. Similarly, human perceived motion of Gabor elements in the collinear direction was found to move faster than in the orthogonal direction^50^,^52^. Thus, the results from the above studies may suggest the existence of a temporal component that influences the perception of the COL vs ISO-CROSS configurations.

Therefore, in this study we wanted to determine whether the ISO-CROSS phenomenon can be explained by spatial and temporal properties. We hypothesized that there are both spatial and temporal components causing the abolished facilitation in the ISO-CROSS configuration. The spatial component may be caused by 1) an interaction between the SBS and the COL flankers. 2) Since the facilitation is also shown to be associated with a “filling-in” effect,^9^,^29^,^30^,^31^,^32^, it is possible that the noise increases in ISO-CROSS, leading to a decreasing signal-to-noise ratio. 3) We also hypothesized that there is a temporal component, i.e. facilitation revealed by ISO-CROSS may occur due to the propagation time that matches the target’s response, thus resulting in facilitation^8^,^47^, whereas that of the SBS flankers is not matched; thus, it might cause a temporal asynchrony that affects the collinear facilitation.

## Results

**Figure 1:**
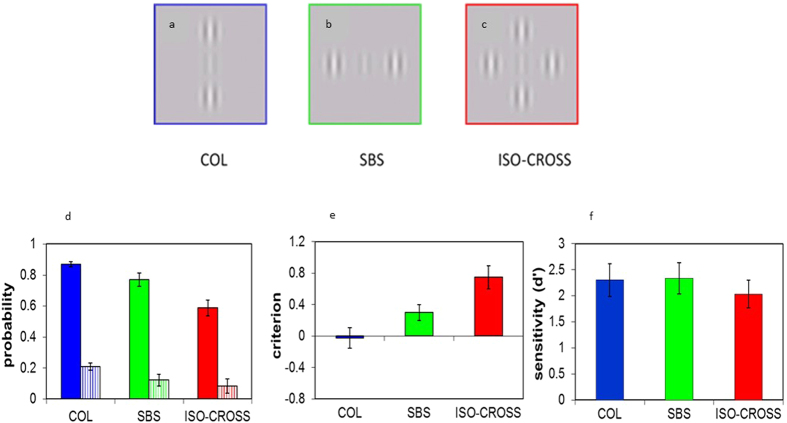
Example of the stimuli and results. Gabor target and flankers positioned at different spatial configurations. (**a**) Collinear (COL); (**b**) side-by-side (SBS); (**c**) COL + SBS producing a cross configuration (ISO-CROSS). The colors of the frame in each stimulus match the color of the relevant bar in figures d–f. (**d**) Bar charts for the probability of hit (solid bars) and false alarm rates; (**e**) decision criterion, and (**f**) sensitivity. Error bars denote the mean ± standard error (N = 14).

We used the Yes/No paradigm^9^,^47^,^59^,^60^ to measure the hit and false alarm rates for the COL, SBS, and ISO-CROSS configurations intermixed by trials. The results are presented in Fig. 1 (N = 14). Figure 1a shows the probability of the hit and false alarm rates, showing that the hit rates for the COL and SBS configurations were significantly higher than for the ISO-CROSS configuration (blue, green, red-filled bars for COL, SBS, and ISO-CROSS, respectively) (COL: p < 0.0000, SBS; p = 0.0002, paired t-test). The false alarm rate (dashed bars) was also significantly higher for the COL than for the ISO-CROSS configuration (p = 0.00004, paired t-test) but was not significantly different between SBS and ISO-CROSS (p = 0.25, paired t-test). When comparing the COL and the SBS configurations, the hit and false alarm rates were significantly higher for COL (hit rate: p = 0.027, false alarm rate; p = 0.041, paired t-test). The decision criteria (Criterion (Cr), Fig. 1b) for the ISO-CROSS configuration is significantly higher than the COL and SBS configuration (COL; p < 0.0000; SBS; p = 0.014, paired t-test) indicating that the subject’s reports are more “target no-present” for the ISO-CROSS. The Cr for COL is significantly lower than for SBS (p = 0.014; paired t-test) indicating that the subject’s reports are more “target present” for the COL. Since Cr was the lowest for COL, and Cr is correlated with the effect of collinear facilitation^9^, the results of the Cr and the hit rate provide support for previous studies showing that facilitation is strongest for COL. Importantly, the fact that the false alarm rate decreased, whereas Cr increased in ISO-CROSS does not support the suggestion that strong excitation from COL and SBS contributes simultaneous signals to the perceptive field^10^,^81^,^82^,^83^ that processes ISO-CROSS; thus, it may be responsible for the cancelation effect of facilitation in ISO-CROSS. In addition, the suggestion of increased noise (filling-in) by ISO-CROSS is not supported, since the false alarm rate is reduced in ISO-CROSS. Figure 1c shows that the sensitivity (d’) is not significantly different among the three spatial configurations (COL *vs* SBS, p = 0.86; COL *vs* ISO-CROSS, p = 0.1; SBS *vs* ISO-CROSS, p = 0.18). This result is consistent with previous studies showing that d’ is not an optimal measure of collinear facilitation when using the Yes/No paradigm^9^,^47^,^59^,^60^. The reasoning for these results was described and discussed previously^9^,^47^.

### Specificity of spatial configuration

We investigated whether the configuration effect of the collinear facilitation observed in Polat and Sagi’s study^9^ and the cancelation effect in ISO-CROSS are due to the positioning of any flankers at the side location or are due to a specific iso-oriented configuration. It is known that lateral facilitation diminishes as the orientation differences between the target and flankers is increased^1^. We performed another experiment by changing the configuration of the SBS flankers from iso-oriented to be orthogonal to the target (ORTO), resulting in control for the side-by-side configuration (ORTO-SBS). Thus, the control experiment mixed the COL, ORTO, and ORTO-CROSS conditions.

**Figure 2:**
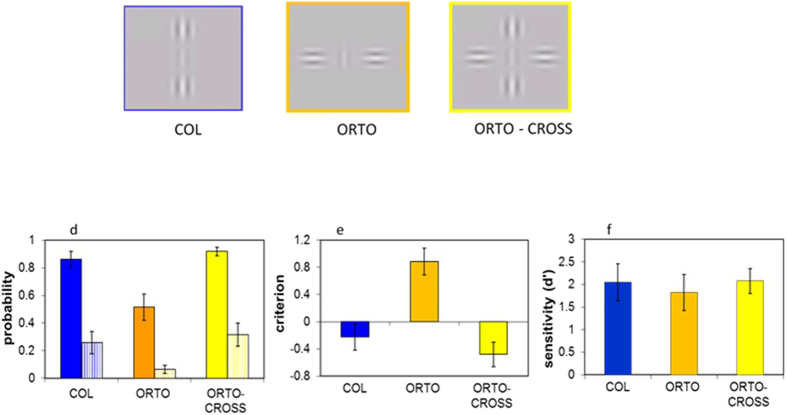
Example of the stimuli and control for the spatial configurations. Gabor target and flankers positioned at different spatial configurations. (**a**) Collinear (COL); (**b**) side-by-side but for an orthogonal configuration of the flankers (ORTO); (**c**) COL + ORTO producing the arto-cross configuration (ORTO-CROSS). The colors of the frame in each stimulus match the color of the relevant bar in figures d–f. (**d**) Probability of the hit rate (filled bars) and false alarms (dashed bars) for the collinear configuration (COL), ORTO), and combined, COL, and SBS producing a ORTO-CROSS configuration. (**e**) Decision criteria (criterion). (**f**) Sensitivity (d’). Error bars denote the mean ± standard error (N = 7).

The results are presented in Fig. 2. The pattern of the results for *ORTO-CROSS* is different from that of ISO-CROSS. Unlike the results presented in Fig. 1d, the results (Fig. 2d) for the hit and false alarm rates of COL and *ORTO-CROSS* are not significantly different (COL vs *ORTO-CROSS*, hit rate; p = 0.096, false alarm rate; p = 0.42). The hit and false alarm rates for COL and *ORTO-CROSS* were significantly higher than for the ORTO configuration (hit rate: p = 0.0003, p = 0.0004; false alarm rate: p = 0.01, p = 0.007; paired t-test, respectively). The Cr (Fig. 2e) for COL is slightly more negative compared with the COL in Fig. 1e, but this difference is not significant. In contrast, the Cr for ORTO (Fig. 2e) is significantly higher (more “no” answers) than the Cr for the SBS (Fig 1b) (p = 0.017; unequal sample t-test), suggesting that the orthogonal flanker induces a suppressive effect^32^,^33^,^60^. The Cr for COL and for *ORTO-CROSS* are significantly lower than the Cr for ORTO (p < 0000, p < 0.0000, paired t-test, respectively). There is no significant difference between the Cr of COL and that of *ORTO-CROSS* (p = 0.13 paired t-test). The results of d’ are not significantly different among the spatial configurations (Fig. 2f). Thus, the results of this control experiment indicate that the cancellation effect that is described in Fig. 1 is configuration dependent, occurring only for iso-orientations, as expected from the architecture of the collinear facilitation^1^,^7^.

### Temporal properties – forward presentation of flankers

**Figure 3:**
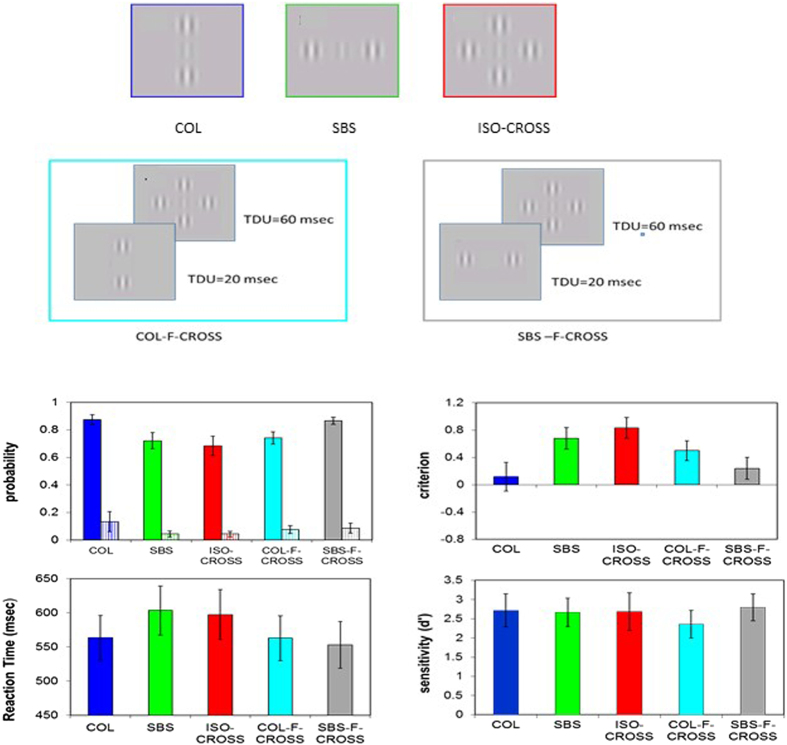
Example of the stimuli and results of the control experiment for the temporal control Gabor target and flankers positioned at different spatial configurations. Collinear (COL); side-by-side (SBS) COL + SBS resulting in an iso-cross configuration (ISO-CROSS). Collinear flankers (COL) presented 20 msec before the ISO-CROSS; SBS flankers presented 20 msec before the ISO-CROSS. The colors of the frame in each stimulus match the color of the relevant bar in the following figures. Probability of the hit rate (filled bars) and the false alarms (dashed bars), Decision criteria (criterion), Reaction time, Sensitivity, Error bars denote the mean ± standard error (N = 7).

We hypothesize that the propagation time of the SBS flankers is slower than that of the COL flankers. Our previous pilot study, using measurements of event-related potential in humans, showed that presenting SBS flankers 20 msec before ISO-CROSS is optimal for restoring the collinear facilitation^84^. Thus, in the next experiment we presented the COL (COL-F-ISO-CROSS) and the SBS flankers (SBS-F-ISO-CROSS) 20 msec before the appearance of the ISO-CROSS condition (forward masking) and they remained presented with ISO-CROSS for another 60 msec. The regular COL, SBS, and ISO-CROSS conditions were also tested (a presentation time of 60 msec); thus, we tested five conditions mixed-by-trial. We also measured the reaction time in this experiment. The results are presented in Fig. 3. The hit rate for the COL configuration is significantly higher than for the SBS (p = 0.018) and ISO-CROSS (p = 0.018); however, the results for false alarm rate are not significantly different (p = 0.13, p = 0.16, respectively). In addition, there is no significant difference between SBS and ISO-CROSS for hit and false alarm rate (p = 0.58, p = 0.97, respectively). Taken together, these results are similar to those presented in Fig. 2d. Likewise, the Cr (Fig. 3b) is lower for SBS and ISO-CROSS (p = 0.031 and p = 0.02, respectively, paired t-test). The Cr is not significantly different between SBS and ISO-CROSS (p = 0.3).

However, a remarkable change occurs when the flankers are presented before ISO-CROSS. Forward presentation of COL flankers 20 msec before ISO-CROSS (COL-F-ISO-CROSS) only slightly increased the hit rate but it did not change it significantly (hit rate: p = 0.17; false alarm rate p = 0.55). In contrast, presenting the SBS flankers (SBS-F-ISO-CROSS) 20 msec before CROSS dramatically and significantly changed the results for the CROSS; the hit rate increased significantly from 0.68 to 0.86 (p = 0.0002, paired t-test); the Cr also decreased significantly from 0.83 to 0.24 (p = 0.011, paired t-test). As a result, the effect of cancelation disappear; the hit rate of the ISO-CROSS configuration, when the SBS presented before, was not significantly different from the COL configuration (p = 0.16). As in the previous experiments, the results for d’ were not significantly different among the configurations. Thus, the results of this control experiment, showing that placing SBS flankers before ISO-CROSS (SBS-F-ISO-CROSS), revealed the expected collinear facilitation. This result supports our hypothesis that a temporal component is involved in the processing of ISO-CROSS. Recent studies measuring intracellular recordings provide support for these results^22^,^23^.

In this experiment we also measured the reaction time to estimate the processing speed of each configuration (Fig. 3). The reaction time is significantly faster (40 msec) for the COL configuration than for the SBS configuration (p = 0.019) and 34 msec faster than the ISO-CROSS configuration (p = 0.001, paired t-test, respectively). There is no significant difference between SBS and ISO-CROSS (p = 68). The reaction time is also faster by 40 msec for the COL-F-ISO-CROS and SBS-F-ISO-CROSS than for ISO-CROSS (p = 0.035, p = 0.01, respectively). Thus, these results further support our hypothesis that COL is processed faster than SBS, consistent with previous data showing that the perception of apparent motion in the collinear direction is perceived as faster than SBS^52^.

## Discussion

We found that the hit rates of the collinear configuration are higher than those of the side-by-side configuration. This result is consistent with the configuration preference of the facilitation to collinear configuration showing lower thresholds^7^, improved threshold summation^85^, synaptic facilitation^22^, and higher brain signals^74^. Here we also confirmed previous results that adding additional flankers to the sides (SBS) of the collinear configuration abolished the superiority of the collinear effect in the ISO-CROSS configuration.

### Spatio-temporal model based on the properties of excitation and inhibition

As mentioned in the Introduction, lateral interactions are both excitatory (*E*) and inhibitory (I)^1^,^8^,^13^,^14^,^15^,^16^,^17^,^18^,^19^,^20^,^21^,^22^. Results suggest that the contextual effects are mediated by the long-range horizontal connections formed by pyramidal neurons within V1^17^,^18^,^19^,^22^,^26^,^27^,^28^,^49^. The emerging results from these studies indicate that excitation is more selective and is received between non-overlapping neurons connected by long-range connections, and have similar optimal orientation selectivity that tends to make preferred connections along the collinear configurations. The inhibitory effect resulted either from the lateral interactions or from local interactions thus is being less selective.

**Figure 4:**
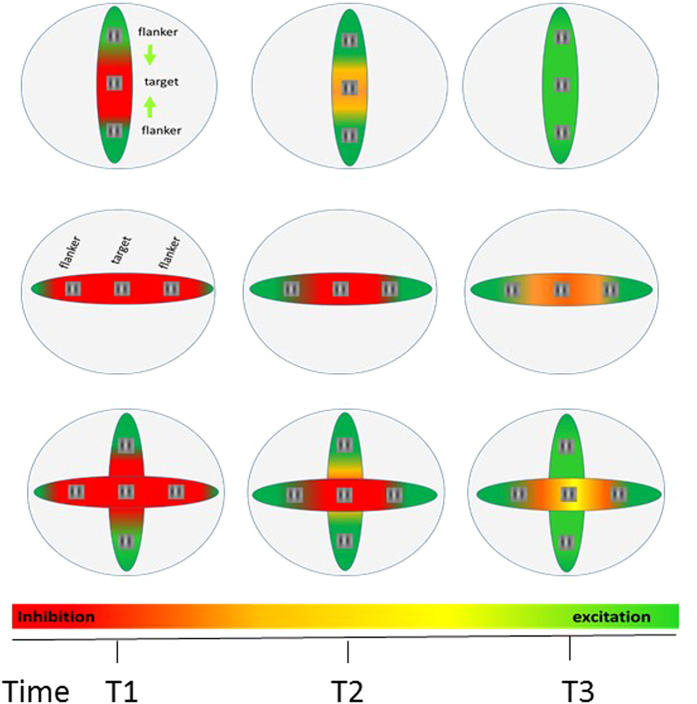
Model illustration of the spatial temporal properties of the excitation and inhibition underscore the lateral interactions. Gabor target and flankers positioned at different spatial configurations producing collinear (COL, top), side-by-side (SBS, middle), and COL + SBS, producing iso-cross configuration (ISO-CROSS, bottom). Each row depicts different time intervals of the lateral propagation, immediate (T1), intermediate (T2), and after the excitation arrives to the target location (T3). The color code indicates that a lateral inhibition that is fast produces suppression (red), whereas a lateral excitation that arrives later produces facilitation (green). Intermediate colors (orange and yellow indicate the balancing effect of the inhibition and excitation, revealing no effect).

In the temporal domain, it was shown that the time constant of the inhibition is rapid and transient^5^,^8^,^14^,^22^. In contrast, the time constant of the excitation is relatively delayed and sustained^8^,^13^,^14^,^20^,^86^,^87^ and may abrogate the inhibition with increasing presentation times^8^,^47^. Recent research using intracellular recordings^22^ show that subthreshold responses to oriented stimuli flashed outside the receptive field exhibited a geometrical organization around the preferred orientation axis, mirroring the psychophysical association field for collinear contour perception; however, non-collinear direction may produce fast shunting inhibition. Thus, the final outcome of the E/I depends on the spatio-temporal parameters determined by the spatial configuration of the target and flankers, the spacing between them, the contrast of both the target and flankers, and the temporal presentation of the target and flankers (see Fig. 4 for an illustration of the model). In this regard, studies from Fregnac and colleagues^22^,^23^ are highly relevant, showing a temporal advantage of about 20 msec of collinear configuration over the parallel configuration.

Previous studies that explored the effect of spatial configurations on the collinear facilitation found that adding additional flankers to the sides of the collinear configuration cancelled the facilitatory effect^10^,^35^. This effect was surprising and challenged previous models of spatial vision. There were a few accounts that attempted to explain this effect. One was that the SBS flankers may increase the excitation^35^, which then shifts the target’s activity from near threshold to a no facilitation zone^10^,^15^,^33^,^57^,^81^,^82^,^83^. If so, one would expect to find an increase in the hit and false alarm rates in ISO-CROSS. An increase in the false alarm may contribute to higher noise^62^; thus, it may cancel the facilitatory effect. Here we found an opposite effect, a decrease in the hit and false alarm rates in ISO-CROSS compared with COL. We also found that Cr is increased in ISO-CROSS, suggesting that the level of neural interaction is shifted to a lower facilitation level^9^. Therefore, an explanation based on the spatial arrangement of SBS contributing to higher excitation is less likely. Another possibility is that the signal from the SBS flankers contribute shunting inhibition that cancel the COL excitation^22^.

An alternative possibility is based on the temporal properties of excitation and inhibition that were described above (see also Fig. 4). If the effect is due to temporal differences between COL and SBS, and the propagation of SBS is slower than that of COL, then when they are combined in the ISO-CROSS configuration, temporal asynchrony similar to backward masking should occur^88^,^89^. Backward masking cancels the collinear facilitation, leading to a reduction of the target’s visibility^88^,^89^ and reduced hit and false alarm rates that should decrease Cr (less target present reports)^88^. It is possible that the early activation of side-band inhibition (advanced by 20 msec in SBS-F-CROSS) has an earlier suppressive (shunting) effect that decays when the delayed excitation provided by the collinear flankers (COL) arrives. Thus, in ISO-CROSS, slower propagation of the SBS signal can contribute to shunting inhibition^22^ that cancels the effect of collinear facilitation. We noted that one of the leading models for backward masking is fast inhibition (similar to shunting inhibition) produced by the mask on the target^8^,^89^,^90^. Indeed, here we found a behavioral effect similar to backward masking, in ISO-CROSS, showing decreased hit and false alarm rates and increased Cr compared with COL and SBS configurations, consistent with what is expected from backward masking^89^. Since the control experiment showed an opposite effect for the control CROSS (ORTO-CROSS), this result supports the idea that the effect is not solely due to the spatial presentation of flankers on the sides of the collinear configuration—it is also due to the temporal property that they may produce fast (shunting) inhibition.

Moreover, we tested the prediction of a temporal asynchrony. When we presented the SBS flankers 20 msec before ISO-CROSS, the effect of cancelation disappeared. This is in agreement with the idea that the propagation time of the COL signals is faster than that of the SBS flankers^50^,^52^. Thus, SBS signals, arriving at a slight delay, produce a temporal asynchrony. Support for our prediction of a temporal advantage of COL is found in the reaction time data showing that the reaction time is faster for COL than for SBS and ISO-CROSS (see Fig. 3c). Additional support is found in our pilot event related potential (ERP) study^84^, showing that the latency of SBS is larger than that of COL and that presenting the SBS 20 msec before ISO-CROSS recovers the facilitation in ISO-CROSS. These results are consistent with previous results showing an advantage of the collinear response over the non-collinear configuration^7^,^31^,^35^,^50^,^52^,^74^,^85^; this is supported by recent studies by Fregnac and colleagues^22^,^23^.

It was suggested that temporal information can lead to segregation of objects from their background with a time difference within 5 msec^67^,^68^,^69^,^70^,^71^. Results indicate that a small temporal asynchrony, below the visual integration timescale, can have a direct effect on the grouping^73^. The results also provide psychophysical and computational support, suggesting that the visual system implements a mechanism that synchronizes the response onsets to object parts and attenuates or cancels their latency differences^72^. It was largely assumed that collinear interactions serve as mechanisms involved in grouping contour elements^7^,^33^,^35^,^74^,^75^,^76^,^77^,^78^. This suggestion was backed by data showing that under collinear facilitation the neural response variance decreased^79^, which improved the temporal correlation between the contour elements^80^. However, our results show that adding the SBS flankers to COL abolished the collinear facilitation, suggesting that the temporal delay of SBS is larger than the time delay that the visual system can attenuate^72^. Thus, our data suggest that a temporal asynchrony larger than a few msec cannot be attenuated to support grouping.

It is largely assumed that masking and crowding are different^63^,^64^,^91^. Several studies, using a set of Gabor patches, showed that global configuration affects crowding^66^,^92^,^93^,^94^. The general view from these studies is that crowding is more pronounced when the effect of grouping increases. Related to our study is the use of the ISO-CROSS configuration^66^ as a stimulus for exploring crowding, which showed that ISO-CROSS impairs orientation discrimination. Our recent results^47^ suggest that under certain spatiotemporal conditions, visual crowding and masking share common neural mechanisms of excitation and inhibition. Here we kept the spatial separation constant (3λ), at a range that is known to reveal facilitation, and we showed that changing the global configuration abolished the perception of collinear facilitation. Therefore, the effect of ISO-CROSS can be seen as crowding, despite that the target is at the threshold and the task is detection. In other words, since the E/I level for facilitation depends on the stimulus parameters and on the network properties, the delayed signal from SBS may contribute to inhibition by shifting the network to a level where there is no facilitation.

It was suggested that collinear facilitation provides an advantage of functional significance^7^,^9^,^22^,^31^,^33^,^35^,^74^,^85^,^95^,^96^ that may contribute to the assigning of an image’s contours, providing a substrate for further cognitive processing. This experimental result is also supported by the statistics of natural images^97^. Thus, one would expect that the faster response (of COL) should prevail in the ISO-CROSS configuration. However, surprisingly, we saw that a slower response of SBS modifies the perception of collinear facilitation that is widely believed to play a functional role in contour integration. The change in this perception may therefore suggest another perceptual significance of ISO-CROSS (and crowding) in the processing of surfaces and textures.

## Methods

### Subjects

Twenty-one subjects with normal or corrected-to-normal visual acuity participated in the experiments. The procedures were approved by the ethics committee of the Sheba Medical Center and all participants gave informed written consent to participate in the study. All experimental protocols were performed in accordance with the guidelines provided by the committee approving the experiments.

### Apparatus

Stimuli were displayed as gray-level modulation on a Philips 107P color monitor. The experiments were controlled by a Dell PC. Screen resolution was 1024 × 768 pixels occupying a 9.20 × 12.20 of visual degrees. The refresh rate was 100 Hz. The mean display luminance was 40 cd/m2 in an otherwise dark environment. Gamma correction was applied. The stimuli were viewed from a distance of 150 cm.

### Visual stimuli

The stimuli were presented as gray-level images (Gabor patches) with a spatial frequency of 6 cycles per degree (cpd) modulated from a background luminance of 40 cd/m^2^, with a 60 msec duration. The spread of the Gaussian envelope (б) was equated with the wavelength (λ, 0.166°) of the carrier^1^. The target’s contrast was adjusted to the participant’s threshold (4–7%) and the contrast of the flankers was always 60%. The target-flanker separation was always 3λ for all spatial configurations: collinear (COL), side-by-side (SBS), and a combination of COL and SBS producing ISO-CROSS (Fig. 1). The orientations of the target and flankers were vertical.

We performed two control experiments: a spatial control experiment in which the side-by-side flankers were orthogonal to the target (ORTO), which resulted in control for ISO-CROSS (ORTO-CROSS). We also performed temporal control experiment in which the COL or the SBS flanker was presented 20 msec before ISO-CROSS (COL-F-ISO-CROSS, SBS-F-ISO-CROSS) and remained presented as part of ISO-CROSS for the remaining 60 msec. Thus, in this experiment five conditions were mixed-by-trial.

### Experimental procedures

A Yes/No paradigm was used^9^,^47^,^59^,^60^,^61^. Subjects were asked to detect a target that may appear or not (Yes/No) between the flankers in all different configurations (COL, SBS, ISO-CROSS, ORTO, and ORTO-CROSS). Target and non-target trials appeared randomly. Participants reported whether the target was present (Yes) or absent (No) by pressing the left and right mouse keys, respectively. They were informed of a wrong answer by auditory feedback after each presentation throughout the experiment. A visible fixation circle appeared in the center before each trial and disappeared when the trial started. The order of the configurations was randomized between trials (the “Mix” procedure); each orientation was presented 100 times per configuration.

The false alarm, miss, hit, and correct rejection rates were recorded and analyzed to yield the sensitivity (d’ = z(Hit)-z(FA)) and the criterion (Cr = (z(Hit) + z(FA))/2) measures, with z defined as the inverse of the normal distribution function. This calculation was used in the previous studies^9^,^47^,^59^,^60^,^61^ and is based on MacMillian and Creelman’s equation^98^, which can be viewed as a deviation from the ideal observer’s decision criterion. The experiments were performed using the “Mix” procedure^9^,^47^,^59^,^60^. In the “Mix” procedure, the trials with different target–flanker configurations are presented in a random order. Each configuration was presented 100 times with the target present in about half of the trials (a probability of 0.5).

## Additional Information

**How to cite this article**: Lev, M. and Polat, U. Temporal asynchrony and spatial perception. *Sci. Rep.* **6**, 30413; doi: 10.1038/srep30413 (2016).
